# A prediction model integrating synchronization biomarkers and clinical features to identify responders to vagus nerve stimulation among pediatric patients with drug‐resistant epilepsy

**DOI:** 10.1111/cns.13923

**Published:** 2022-07-27

**Authors:** Jiayi Ma, Zhiyan Wang, Tungyang Cheng, Yingbing Hu, Xiaoya Qin, Wen Wang, Guojing Yu, Qingzhu Liu, Taoyun Ji, Han Xie, Daqi Zha, Shuang Wang, Zhixian Yang, Xiaoyan Liu, Lixin Cai, Yuwu Jiang, Hongwei Hao, Jing Wang, Luming Li, Ye Wu

**Affiliations:** ^1^ Department of Pediatrics Peking University First Hospital Beijing China; ^2^ National Engineering laboratory for Neuromodulation, School of Aerospace Engineering Tsinghua University Beijing China; ^3^ Pediatric Epilepsy Center Peking University First Hospital Beijing China; ^4^ Beijing Key Laboratory of Epilepsy Research, Department of Neurology, Center of Epilepsy, Beijing Institute for Brain Disorders, Sanbo Brain Hospital Capital Medical University Beijing China; ^5^ Precision Medicine & Healthcare Research Center, Tsinghua‐Berkeley Shenzhen Institute Tsinghua University Shenzhen China; ^6^ IDG/McGovern Institute for Brain Research Tsinghua University Beijing China; ^7^ Institute of Epilepsy Beijing Institute for Brain Disorders Beijing China

**Keywords:** drug‐resistant epilepsy, machine learning, scalp electroencephalography, synchronization, vagus nerve stimulation

## Abstract

**Aims:**

Vagus nerve stimulation (VNS) is a neuromodulation therapy for children with drug‐resistant epilepsy (DRE). The efficacy of VNS is heterogeneous. A prediction model is needed to predict the efficacy before implantation.

**Methods:**

We collected data from children with DRE who underwent VNS implantation and received regular programming for at least 1 year. Preoperative clinical information and scalp video electroencephalography (EEG) were available in 88 children. Synchronization features, including phase lag index (PLI), weighted phase lag index (wPLI), and phase‐locking value (PLV), were compared between responders and non‐responders. We further adapted a support vector machine (SVM) classifier selected from 25 clinical and 18 synchronization features to build a prediction model for efficacy in a discovery cohort (*n* = 70) and was tested in an independent validation cohort (*n* = 18).

**Results:**

In the discovery cohort, the average interictal awake PLI in the high beta band was significantly higher in responders than non‐responders (*p* < 0.05). The SVM classifier generated from integrating both clinical and synchronization features had the best prediction efficacy, demonstrating an accuracy of 75.7%, precision of 80.8% and area under the receiver operating characteristic (AUC) of 0.766 on 10‐fold cross‐validation. In the validation cohort, the prediction model demonstrated an accuracy of 61.1%.

**Conclusion:**

This study established the first prediction model integrating clinical and baseline synchronization features for preoperative VNS responder screening among children with DRE. With further optimization of the model, we hope to provide an effective and convenient method for identifying responders before VNS implantation.

## Graphical abstract

A support vector machine prediction model integrating clinical and baseline synchronization features was established for preoperative screening of responders to vagus nerve stimulation among children with drug‐resistant epilepsy.

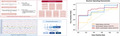

## 1 INTRODUCTION

Vagus nerve stimulation (VNS) is a neuromodulation therapy for drug‐resistant epilepsy (DRE) in both children and adults. VNS was first approved by the U.S. Food and Drug Administration for patients with DRE in 1997, and its efficacy and safety have been proven for over 20 years.^1^ A recent meta‐analysis including 101 pediatric studies found that approximately 56.4% of children with DRE achieved ≥50% seizure reduction (defined as responders) compared to the pretreatment baseline, and 11.7% of children were seizure‐free at the last follow‐up (>1 year).^2^ Although VNS significantly decreases the costs related to hospitalization, anti‐seizure medications (ASMs), and other costly downstream healthcare interventions in responders,^3^, ^4^ the total expense of VNS treatment is still a heavy financial burden for families, especially in developing countries, with costs ranging from EUR 65 to 24,790.^5^, ^6^ The average expense for the pediatric population is approximately 1.7 times higher than that of the general population.^7^ Furthermore, despite advancements in surgical procedures, children still inevitably need to undergo general anesthesia and undertake the risks of any invasive surgery. As a consequence, taking into account the heterogeneity in efficacy, it is essential to identify potential candidates who are more likely to benefit from VNS in advance to avoid unnecessary implantation. A high specificity and convenient prediction model applicable for identifying pediatric responders before VNS implantation in clinical practice is in crucial need.^8^, ^9^, ^10^, ^11^, ^12^, ^13^

Although the exact antiepileptic mechanisms of VNS remained unclear, desynchronization recorded by electroencephalogram (EEG) was considered to be a possible mechanism as VNS inducing desynchronization in specific frequency band was proved in both animal and human researches.^14^, ^15^, ^16^ Based on the underlying desynchronization mechanism, synchronicity indexes extracted from EEG, including the phase locking value (PLV), phase lag index (PLI) and weighted PLI (wPLI), were found to be related to postoperative VNS efficacy.^16^, ^17^, ^18^, ^19^, ^20^ Sangare et al.^20^ reported a significant correlation between lower PLI OFF/ON ratio in delta, theta and beta bands at awake state and better VNS efficacy in 35 patients with DRE. Vespa et al.^16^ found that stronger VNS‐induced theta desynchronization correlated with better clinical outcome and wPLI OFF/ON ratio in theta band could be considered as a biomarker to identify responders from non‐responders. In theory, the variability of synchronicity index might reflect the integrity of individual functional brain network connectivity, which responders showed significant desynchronization receiving external stimulation compared to non‐responders. Therefore, we speculate that epilepsy patients with higher preoperative synchronicity indexes might be potential VNS responders.

With the wide application of machine learning, researchers have adopted relevant methods for predicting therapeutic efficacy.^21^, ^22^, ^23^ Support vector machine (SVM), which is a binary classification model, is widely used in epileptic field, including prediction of epilepsy surgery outcomes, patient‐specific seizure prediction and epilepsy diagnosis based on a small dataset.^21^, ^22^, ^24^ As Mithani et al. conducted SVM prediction model, which indicated the unreliability based on clinical phenotypes only, the accuracy of the prediction algorithm was likely to be improved when holding the major factors and eliminating the minor factors by SVM.^11^, ^12^, ^13^ On these grounds, we explored a prediction SVM model integrating clinical and synchronization features extracted from scalp EEG before implantation, with the aim of predicting postoperative VNS efficacy in children with DRE.

## 2 METHODS

### 2.1 Patients and clinical information

#### 2.1.1 Inclusion criteria

We collected data from children with DRE who underwent implantation of a vagus nerve stimulator (PINS, Beijing, China or Cyberonics, Houston, TX) between March 2016 and December 2020 from 2 separate epilepsy centers: Peking University First Hospital (Beijing, China) and Sanbo Brain Hospital (Beijing, China). Data from patients implanted between March 2016 and June 2020 in Peking University First Hospital were used as cohort for prediction SVM building (discovery cohort). Additional data from patients implanted with VNS from Peking University First Hospital and Sanbo Brain Hospital were used as independent data for validation of the prediction model (validation cohort). Patients included in this study fulfilled all the following criteria: (1) age at implantation ≤16 years; (2) diagnosis of drug‐resistant epilepsy according to the criteria defined by the International League Against Epilepsy (ILAE)^25^; (3) regularly programmed and followed‐up for at least 1 year after implantation; (4) output current ≥1 mA during program; and (5) with preoperative scalp video‐EEG recorded for at least 4 hours (duration of awake state ≥40 min) within 3 months prior to implantation available. Patients who received newly added antiepileptic therapy after VNS (including surgical treatment, ketogenic diet, and other anti‐seizure medications), terminated VNS therapy, lost to follow‐up or with irregular/absent seizure frequency recordings were excluded.

#### 2.1.2 Baseline clinical information

Baseline clinical information included gender, body mass index (BMI), diastolic and pulse pressure, age at onset, age at implantation, duration of epilepsy before surgery, seizure type, frequency of seizures, etiology of epilepsy, history of any previous epilepsy surgery or diagnosis of developmental and epileptic encephalopathies (DEEs),^26^ number of historical anti‐seizure medications (ASMs) used, number of ASMs at baseline and brain magnetic resonance imaging (MRI).

#### 2.1.3 Seizure outcome measures

The efficacy outcomes of individual patients were calculated as the average reduction in seizure frequency within 6 months at the last follow‐up compared to baseline (average seizure frequency within 3 months before implantation) according to the epilepsy diary. Children with 100%, ≥80% and ≥50% reductions in seizure frequency were defined as 100% (R100), 80% (R80) and 50% (R50) responders, respectively. Children with a reduction in seizure frequency < 50% were defined as non‐responders (NR50).

### 2.2 Scalp video‐EEG recording and synchronization‐based measures

#### 2.2.1 Scalp video‐EEG recording and processing

Baseline scalp video‐EEG was acquired within 3 months before VNS. Scalp video‐EEG was recorded using a 21‐channel EEG system (Neurofax, EEG‐1200C) positioned according to the 10–20 system placement (Fp1, Fp2, F3, F4, C3, C4, P3, P4, O1, O2, T3, T4, T5, T6, Fz, Cz, Pz, F7, and F8).

EEG processing was carried out in MATLAB R2018b (Mathworks), using EEGLAB (2020).^27^ For each patient, at least 6 min of awake interictal EEG data (identified by synchronized video or eye movement artifact) were selected. For better implementation of data stationarity in subsequent processing, selected data was further analyzed into epochs with 2 s length each. Epochs with apparent eye movement artifacts or epileptiform discharges were removed by visual analysis. Then, signals were digitized at a sampling rate of 500 Hz. Bandpass filtering was applied in standard EEG frequency bands: delta (1–4 Hz), theta (4–8 Hz), alpha (8–13 Hz), low beta (13–20 Hz), high beta (20–29 Hz) and beta (13–29 Hz).

#### 2.2.2 Synchronization‐based measures: Phase‐locking value (PLV), Phase lag index (PLI) and Weighted phase lag index (wPLI)

Traditionally, EEG analysis indices based on coherence could represent only the linear dependence between signals because they could not exclude the influence of the amplitude and phase of EEG signals. Therefore, Lachaux et al.^28^ proposed that the PLV could be used to evaluate the phase synchronization degree between signals. We performed a Hilbert transform on the preprocessed whole time series EEG signals to obtain the instantaneous phase time series of EEG signals at each electrode. The PLV was calculated by the phase difference between different signals, and the PLV range was [0,1]. When the PLV was equal to 1, the phase difference time series was constant throughout the whole time series. If the PLV is equal to 0, it means that the phase difference is evenly distributed within the range of (0,2π). The mean global PLV was calculated by averaging PLV values from bipolar EEG channels. Mean global interictal PLV values in awake state were compared for each frequency band between the R50 and NR50 groups.

Although the PLV is widely used, it has an obvious disadvantage: it is sensitive to the common source problem; that is, it is susceptible to the volume conduction. Therefore, Stam et al.^29^ proposed that the PLI could be used to accurately evaluate the phase synchronization degree between different signals. The PLI evaluates the asymmetry of the distribution of phase differences between signals. Similarly, we calculated the phase difference between different EEG signals as we did in the calculation of the PLV and further calculated the value of the PLI in the range of [0, 1]. When the PLI value is 1, there is a constant phase difference, and it is not 0 or π. When the PLI value is 0, there is no connectivity between the EEG signals. The mean global PLI was calculated by averaging PLI values from bipolar EEG channels. Mean global PLI values in awake state were compared for each frequency band between the R50 and NR50 groups.

The wPLI is a phase‐based connectivity evaluation index proposed by Vinck et al.^30^ which is used to improve the discontinuity of PLI indicators. We performed cross‐spectrum analysis of different EEG signals and then further calculated the wPLI values. The wPLI ranges from 0 to 1. Higher wPLI values mean higher connectivity between EEG signals. The mean global wPLI was calculated by averaging the wPLI values from bipolar EEG channels. Mean global wPLI values in awake state were compared for each frequency band between the R50 and NR50 groups.

### 2.3 SVM learning and prediction model

#### 2.3.1 SVM learning

A prediction model for efficacy was built in the form of a linear SVM, which is a frequently applied supervised machine learning method. The efficacy of VNS in the discovery cohort, which was set to be the expected outcome of the prediction, was labeled R50s or NR50s. A total of 43 features (25 clinical and 18 synchronization features) were included in the model as input to generate a binary linear classifier. Twenty‐five clinical features included BMI, diastolic pressure, pulse pressure, age of VNS implantation, duration of epilepsy before implantation, age of seizure onset, history of epilepsy surgery, seizure frequency, etiology of epilepsy (including structural, autoimmune, genetic and unknown), seizure type (including generalized, focal, spasms and multiple types), epilepsy syndrome (including infantile spasms, Lennox–Gastaut Syndrome, early‐onset epileptic encephalopathy and unclassified syndrome), brain MRI (including local, multifocal and negative), number of historical ASMs usage and number of ASMs at baseline. And 18 synchronization features included the PLV, PLI, and wPLI calculated in six bandwidths (delta, theta, alpha, beta, low beta and high beta).

To build a more comprehensive model and improve the performance, redundant or irrelevant features were eliminated through feature selection methods. We applied the filter method with the F‐score and the wrapper method with recursive feature elimination (RFE) in this work. The filter method works in a way that sorts all the features by a specific statistical method and selects a certain number of features that rank at the top. Unlike the filter method, the wrapper method takes advantage of the learning performance of a model to assess the features, which means that its performance is dependent on the learning algorithm itself but may result in a more complicated and slower calculation. We applied both methods to compare their performance.

The nested cross‐validation (nested‐CV) method was carried out during model building. The CV method includes two loop layers. The inner loop aimed to find the best parameters of the model, while each fold of the outer loop provides the validation performance of the model independently by calculating an estimate of error. A 5‐fold CV method was carried out in the inner loop, while both 5‐fold and 10‐fold CV method was tested in the outer loop. For decreasing the overfitting problems to maximum extent, we finally applied a 10‐fold CV method to the outer loop. The performance of the model was assessed through the confusion matrix and receiver operating characteristic curve (ROC curve). Accuracy, precision, and area under the ROC curve (AUC) were calculated.

#### 2.3.2 Validation of the model

Completely independent sample data were collected as a validation cohort to validate the prediction model. All the datasets were collected and analyzed in the same way as the discovery cohort, and clinical and synchronization features were also collected according to a standardized process. The prediction SVM model was then applied to predict the responder status of each patient. Meanwhile, a separate researcher calculated the seizure outcome without knowing the prediction result. The final validation result was calculated according to the accuracy.

### 2.4 Statistical analysis

All the continuous variables were analyzed by Kolmogorov Smirnov test to evaluate data distribution. Subject demographics information and clinical characteristics were analyzed using the Mann–Whitney test for continuous variables and chi‐square analysis or Fisher's exact test for nominal data. Electrophysiological synchronization features were compared between groups with unpaired *t* tests in each frequency band, and false discovery rate (FDR) correction was applied to avoid misleading low p‐values.^31^ Statistical analysis and graphing were performed using MATLAB 2018b and GraphPad Prism 8.0. A significance level of 0.05 was set.

## 3 RESULTS

### 3.1 Baseline clinical features were comparable between responders and non‐responders

**FIGURE 1:**
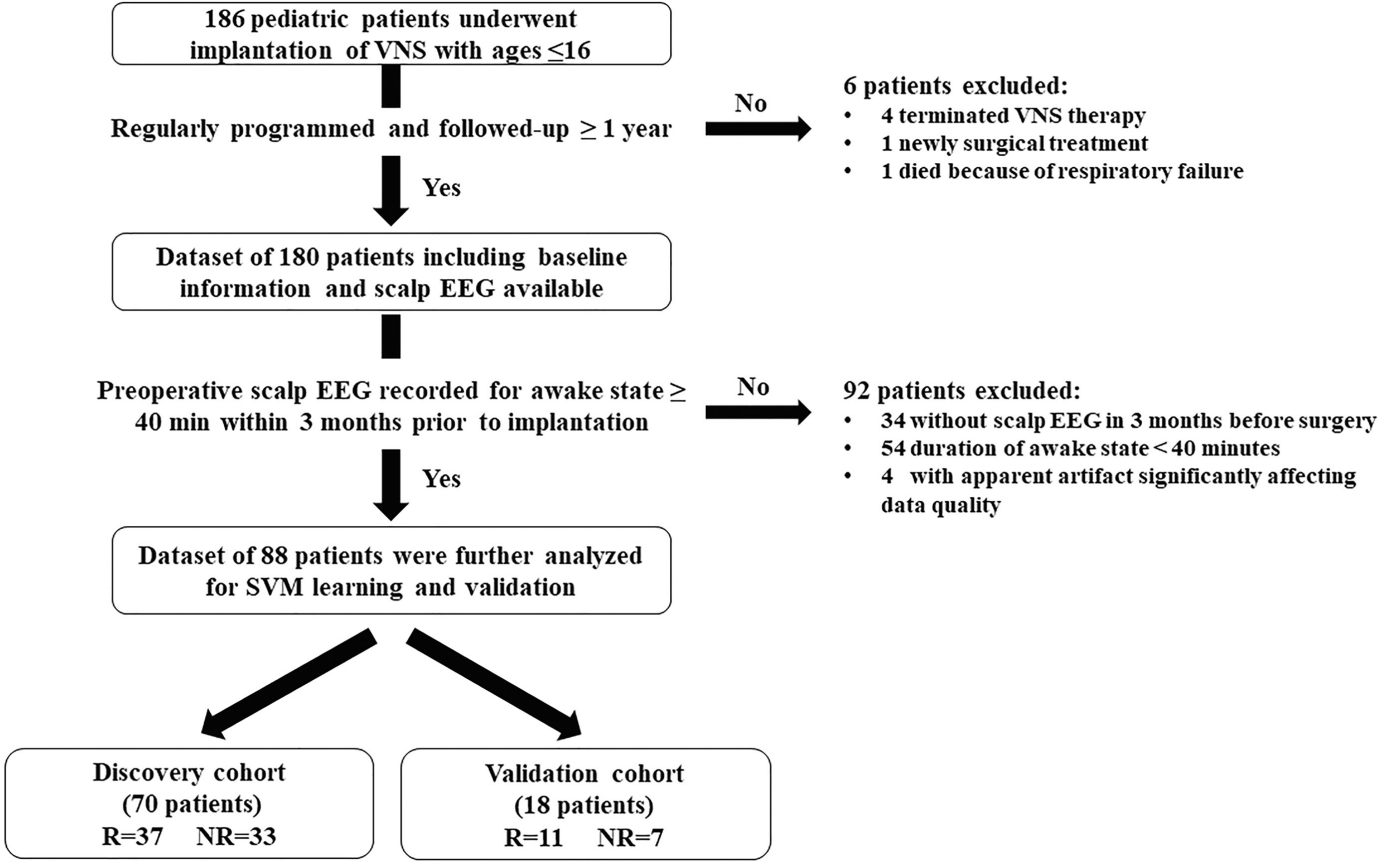
Flow chart of the study design A total of 88 patients met the inclusion criteria for our study, among whom 70 children were further randomly selected for the discovery cohort, while an independent cohort of 18 children was selected for the validation cohort. DRE, drug‐resistant epilepsy; EEG, electroencephalography; NR, non‐responders; R, responders; SVM, support vector machine.

Eighty‐eight children (54 males and 34 females; age at VNS implantation ranging 1.8–15.4 years) with DRE were finally included in our study (shown in Figure 1), among whom 70 patients were used as the cohort for building of prediction SVM (discovery cohort), and 18 patients for the validation. A total of 52.9% (37/70) and 47.1% (33/70) of patients were defined as having R50 and NR50 in the discovery cohort, respectively, after VNS treatment for an average of 2.95 years (range 1.22 to 5.58 years). A comparable summary of the baseline clinical features of the R50 and NR50 groups from the discovery cohort is presented in Table 1. No significant difference was found in clinical features between the R50 and NR50 groups at baseline.

**TABLE 1:** Comparison of the baseline clinical features between the R50 and NR50 groups

Baseline variables (*n* = 70)	R50 (*n* = 37, 52.9%)	NR50 (*n* = 33, 47.1%)	*p* Value
Seizure frequency mean ± SD (range), times/month	681.9 ± 1187 (4–5430)	696.7 ± 764.5 (1.5–2490)	0.52^a^
Gender, *n*			
Male	25	19	0.46^b^
Female	12	14	
BMI mean ± SD (range), kg/m^2^	16.4 ± 2.9 (11.8–23.1)	17.1 ± 3.6 (12.7–29.3)	0.78^a^
Diastolic Pressure mean ± SD (range), mmHg	59.8 ± 5.9 (40–73)	59.9 ± 6.6 (47–76)	0.86^a^
Pulse Pressure mean ± SD (range), mmHg	40.8 ± 6.9 (28–58)	41.7 ± 8.0 (21–68)	0.73^a^
Age of seizure onset mean ± SD (range), years	2.3 ± 3.0 (0.0–11.7)	2.1 ± 2.3 (0.0–8.8)	1.00^a^
Duration of epilepsy before implantation mean ± SD (range), years	3.5 ± 1.4 (1.0–7.3)	3.1 ± 2.3 (0.5–10.0)	0.06^a^
Age at VNS implantation mean ± SD (range), years	5.9 ± 3.1 (1.8–15.4)	5.2 ± 3.0 (1.9–15.0)	0.12^a^
History of previous epilepsy surgery, *n* (%)	6 (43.2%)	3 (9.1%)	0.48^b^
Etiology of epilepsy, *n*, (%)			
Structural	20 (54.1%)	19 (57.6%)	0.76^b^
Genetic	2 (5.4%)	1 (3.0%)	
Autoimmune	1 (2.7%)	0 (0.0%)	
Unknown	14 (37.8%)	13 (39.4%)	
Predominant seizure type, *n* (%)			
Generalized	11 (29.7%)	11 (33.3%)	0.51^b^
Focal	17 (46.0%)	16 (48.5%)	
Spasms	24 (64.9%)	15 (45.5%)	
Multiple types	14 (37.8%)	18 (54.6%)	
Epilepsy syndrome, *n* (%)			
Infantile spasms	11 (29.7%)	4 (12.1%)	0.30^b^
Lennox–Gastaut Syndrome	3 (8.1%)	2 (6.1%)	
EOEE	4 (10.8%)	4 (12.1%)	
Unclassified	19 (51.4%)	23 (69.7%)	
MRI, *n* (%)			
Multifocal	19 (51.4%)	17 (51.5%)	0.84^b^
Local^c^	3 (8.1%)	4 (12.1%)	
Negative	15 40.5%)	12 (36.4%)	
Number of ASMs at baseline mean ± SD (range)	2.9 ± 1.0 (0–5)	3.2 ± 0.9 (2–5)	0.27^a^
Number of historical ASMs mean ± SD (range)	5.5 ± 2.2 (2–10)	5.6 ± 1.8 (2–9)	0.67^a^
Usage of benzodiazepines^d^, *n* (%)	20 (54.1%)	14 (42.4%)	0.35^b^

Abbreviations: ASMs, Anti‐seizure medications; BMI, body mass index; EOEE, early‐onset epileptic encephalopathy; MRI, magnetic resonance imaging; NR, non‐responders; R, responders; SD, standard deviation; VNS, vagus nerve stimulation.

^a^ Calculated using nonparametric Mann–Whitney U test.

^b^ Calculated using chi‐squared (χ^2^) test.

^c^ Patients with local findings in brain MRI had undergone preoperative evaluation who were identified as unfitful for resection surgery.

^d^ The types of benzodiazepines used in this study included phenobarbital, clonazepam, nitrazepam and clobazam.

Among 37 patients defined as R50, 27.0% (10/37) and 75.7% (28/37) were further categorized as R100 and R80, respectively. For the VNS parameters at the last follow‐up, the median output currents of the R50 and NR50 groups were 1.7 mA (ranging 1.5–2.25 mA) and 1.7 mA (ranging 1.4–2.0 mA), respectively, and the duty cycle ranged from 10%–38%.

### 3.2 The baseline synchronization features during awake state in scalp EEG differed between responders and non‐responders

**FIGURE 2:**
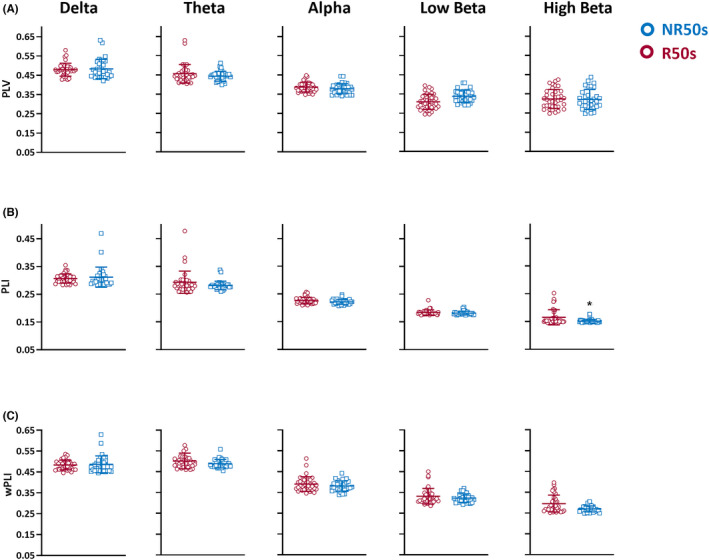
Differences in the PLV, PLI, and wPLI at different frequency bands between the R50 and NR50 groups (A) Differences in the PLV at different frequency bands between the R50 and NR50 groups; there was no significant difference. (B) Differences in the PLI at different frequency bands between the R50 and NR50 groups. The PLI in the R50 group was significantly higher than that in the NR50 group in the high beta band (*p* = 0.045). (C) Differences in the wPLI at different frequency bands between the R50 and NR50 groups. The wPLI in the R50 group was higher than that in the NR50 group in the high beta band, although the difference was not significant (*p* = 0.055). Data are shown as the mean ± SD. All *p* value were applied FDR‐corrected Mann–Whitney U test. NR, non‐responders; PLI, phase lag index; PLV, phase‐locking value; R, responders; wPLI, weighted phase lag index.

The preoperative baseline synchronization features during awake state (including PLV, PLI, and wPLI) were analyzed (Table 2). We found that the PLI in the R50 group was significantly higher than that in the NR50 group in the high beta band (0.165 ± 0.027 vs. 0.152 ± 0.007, *p* = 0.045, FDR‐corrected Mann–Whitney U test, Figure 2B). The PLI and wPLI in the other frequency bands (especially high beta and low beta bands) were also higher in the R50 group, although the difference was not statistically significant. No significant differences in the PLV were found between the R50 and NR50 groups (Figure 2A).

**TABLE 2:** Differences in the PLV, PLI, and wPLI at different frequency bands between the R50 and NR50 groups

	PLV			PLI			wPLI		
	R50s (*n* = 37)	NR50s (*n* = 33)	*p* Value	R50s (*n* = 37)	NR50s (*n* = 33)	*p* Value	R50s (*n* = 37)	NR50s (*n* = 33)	*p* Value
Delta	0.479 ± 0.034	0.483 ± 0.050	0.769	0.307 ± 0.016	0.312 ± 0.036	0.814	0.484 ± 0.024	0.487 ± 0.040	0.656
Theta	0.456 ± 0.048	0.443 ± 0.026	0.769	0.293 ± 0.040	0.281 ± 0.015	0.170	0.501 ± 0.038	0.489 ± 0.020	0.338
Alpha	0.387 ± 0.025	0.378 ± 0.026	0.769	0.226 ± 0.012	0.222 ± 0.009	0.170	0.390 ± 0.035	0.381 ± 0.025	0.640
Low beta	0.341 ± 0.030	0.338 ± 0.032	0.769	0.184 ± 0.010	0.182 ± 0.007	0.513	0.332 ± 0.038	0.323 ± 0.020	0.663
High beta	0.323 ± 0.049	0.321 ± 0.052	0.769	0.164 ± 0.027	0.152 ± 0.007	0.045^a^	0.295 ± 0.041	0.270 ± 0.015	0.055

*Note*: Data was showed in mean ± standard deviation (SD).

Abbreviations: NR, non‐responders; PLI, phase lag index; PLV, phase locking value; R, responders; wPLI, weighted phase lag index.

^a^ *p* < 0.05; All *p* value were applied FDR‐corrected Mann–Whitney U test.

We further evaluated the difference in the wPLI and PLI at the beta bandwidth (low and high beta bands) among R100, R80 and NR50 by subgroup analysis (shown in Table S1). We found that the PLI in the R100 and R80 groups was significantly higher than that in NR50 group in the high beta band (*p* = 0.038 and *p* = 0.006, FDR‐corrected Mann–Whitney U test, respectively). Although there was no statistical significance when comparing the wPLI between the R100 and NR50 groups, higher synchronization was indicated.

Ages significantly affected the background frequency of interictal awake state EEG, especially in high beta bandwidth among younger children. To confirm that the differences in EEG synchronization between R and NR are still applicable to children with different age, patients were categorized into three groups according to age: (1) 0–3 years (*n* = 8); (2) 3–9 years (*n* = 53); and (3) 9–16 years (*n* = 9). A higher wPLI and PLI were still observed at the high beta band in responders in all these groups (shown in Table S2), indicating the constancy of the biomarkers. However, due to the small number of cases in each group, no statistical difference could be reached.

### 3.3 A prediction SVM model integrating clinical and synchronization features to distinguish responders from non‐responders

Three SVM classifiers were generated from the discovery cohort (70 patients) using (1) 25 clinical features, (2) 18 synchronization features and (3) integrating both clinical and synchronization features. We found that the SVM classifier generated from integrating clinical and synchronization features had the best predictive efficiency, with an accuracy of 75.7%, a precision of 80.8% and an AUC of 0.766 on 10‐fold CV (shown in Table 3).

**TABLE 3:** Summary of the accuracy, precision, and AUC values for 3 separate mean 10‐fold cross‐validation‐based SVM learning prediction models in identifying responders/non‐responders in the discovery cohort

	F‐score		
	Accuracy	Precision	AUC
Synchronization features	61.4%	67.5%	0.741
Clinical features	51.4%	60.0%	0.610
Clinical+ Synchronization features	75.7%	80.8%	0.766

Abbreviations: AUC, area under the receiver operating characteristic curve; SVM, support vector machine.

**FIGURE 3:**
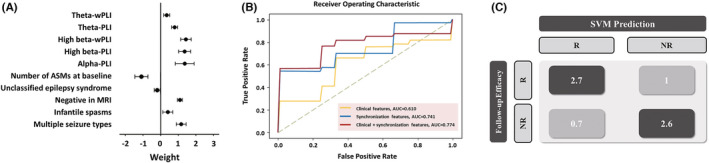
Establishment and validation of the prediction model (A) Ten meaningful principal components selected from 25 clinical features and 18 synchronization features by a support vector machine (SVM) classifier on 10‐fold cross‐validation. Data are shown as the mean ± SD. (B) Receiver operating characteristic (ROC) curve of 3 separate SVM classifiers on 10‐fold cross‐validation. (C) Confusion matrix of the SVM classifier generated from principal component analysis. ASMs, anti‐seizure medications; MRI, magnetic resonance imaging; PLI, phase lag index; wPLI, weighted phase lag index.

From 10 meaningful principal components identified by SVM, the principal component coefficient (mean ± SD) of each was listed below sorted by numerical value: (1) wPLI at high beta band (1.448 ± 0.300); (2) Multiple seizure types (1.308 ± 0.279); (3) PLI at alpha band (1.294 ± 0.533); (4) PLI at high beta band (1.278 ± 0.343); (5) Negative findings in brain MRI (1.190 ± 0.138); (6) PLI at theta band (0.761 ± 0.171); (7) wPLI at theta band (0.331 ± 0.173); (8) Infantile spasms (0.327 ± 0.284); (9) Unclassified epilepsy syndrome (−0.199 ± 0.156); (10) Number of ASMs at baseline (−0.744 ± 0.332). We found that the wPLI in the high beta band, PLI in the high beta band, PLI in the alpha band, multiple seizure types and negative findings in brain MRI had higher principal component coefficients (shown in Figure 3A). The confusion matrix of the SVM classifier generated from principal component analysis was shown in Figure 3C.

The other two SVM classifiers generated from 25 clinical features or 18 synchronization features demonstrated accuracies of 51.4% and 61.4%, respectively, and the AUCs of the ROC curve were significantly lower than those of the ROC curve generated from integrating clinical and synchronization features, as shown in Figure 3B.

The SVM classifier generated from integrating clinical and synchronization features was tested in a validation cohort of 18 patients (10 males and eight females; age at VNS implantation ranging 2.2–15.0 years) from two separate institutions. In this cohort, 11 patients (61.1%) were responders and 7 (38.9%) were non‐responders. The prediction model yielded an accuracy of 61.1%.

## 4 DISCUSSION

To the best of our knowledge, this study is the first to establish a model integrating baseline clinical and synchronization features before implantation through SVM machine learning to predict the efficacy of VNS in children with DRE.

Despite the wide use of VNS in children with DRE, predictive clinical phenotypes and biomarkers for screening potential VNS responders in clinical practice remain unidentified. Clinical phenotypes include later age at onset of epilepsy, shorter epilepsy duration before implantation, particular etiology, seizure type, absence of intellectual disability, fewer ASMs before implantation and so on.^8^, ^9^, ^10^, ^11^, ^12^, ^13^, ^32^ However, none of them could be applied alone in clinical practice due to inconsistencies across studies. Biomarkers mainly focus on electrophysiological activity and network‐based and systemic indexes.^33^ Among these biomarkers, heart rate variability (HRV),^34^ multimodal connectomic prediction algorithm^22^ and genetic variation of adenosine^35^ were reported to be related with long‐term VNS efficacy, with a sensitivity ranging from 9.3%–85.7% and a specificity ranging from 72.4%–100%. However, the study of HRV and genetic variation of adenosine was based on an adult cohort, and it was not tested in a pediatric cohort. The multimodal connectomic prediction algorithm purposed by Mithani et al. demonstrated an accuracy of 89.5%, which was very promising for children with DRE. However, the algorithm requires several tests, including diffusion tensor imaging (DTI) and DTI‐informed magnetoencephalography (MEG), which are complicated, costly, and might not be easily generalizable to clinical practice in children. Hence, the ideal biomarker for prediction should be extracted from data in routine clinical evaluation.

In our study, we applied PLV to give the modulus of the instantaneous phase differences between two time series. Furthermore, to avoid the influence of volume conduction, we applied the wPLI, which is more sensitive in reflecting genuine connectivity changes than the PLI, together with the PLV and PLI in our study.^36^ And the PLI extracted from baseline scalp EEG were significantly different between responders and non‐responders at the high beta band in interictal awake state. Higher frequency band oscillations are also considered to be related to epilepsy and might reflect the beginning of the epileptic network loop.^15^, ^17^ Usami et al.^37^ found that beta oscillations may enhance the responsiveness of cortex to input from distant cortical sites besides gating local cortical processing, indicating the importance of beta oscillations in functional connectivity. Song et al.^38^ reported longer duration of time spent seizure‐free correlated with lower beta power in the seizure onset zone of patients with benign epilepsy with centrotemporal spikes, suggesting beta oscillations might relate with epileptogenicity. When comparing the difference in the PLI at the beta bandwidth in different efficacies (R100 and R80 subgroups) and different age ranges (3–9 years and 9–16 years subgroups), the significance still had good consistency. This result suggested that higher beta oscillations might have better response for VNS and awake PLI in the high beta band is probably potential biomarkers for predicting VNS efficacy. The SVM classifier generated from EEG synchronization features could precisely predict responders with a high AUC value (74.1%), indicating the higher synchronization level at baseline in responders. Interestingly, Mithani et al.^22^ found better left‐lateralized structural and functional connectivity, including the left thalamic, limbic, insular, and temporal regions, in DTI and resting‐state MEG analysis in responders than in non‐responders, which also proved a higher synchronization level in responders from the perspective of structural connectivity before implantation.

Previous studies have fully proven that a more significant decrease in the level of synchronization (including the PLI, wPLI, PLI ratio OFF/ON) during VNS is strongly correlated with better VNS efficacy,^16^, ^17^, ^19^, ^20^ indicating that desynchronization is an important mechanism of VNS.^14^, ^15^, ^39^ Sangare et al.^20^ found that the awake PLI ratio OFF/ON in the beta frequency band correlated with acute VNS efficacy among 35 adult patients. Another study conducted by Bodin et al.^19^ also found that responders had lower awake postoperative global PLI values among 19 patients (14–54 years) who received chronic VNS therapy. In sub‐band PLI analysis, lower frequency bands (delta and alpha) were found to be significantly reduced in 10 responders. Similarly, Vespa et al.^16^ applied the postoperative wPLI OFF/ON ratio of 24 adult patients in the N2 sleep state in the theta band as a potential biomarker to distinguish responders from non‐responders after surgery, yielding an AUC of 0.825. Interestingly, these two studies failed to find a significant difference when focusing on desynchronization in the higher frequency band. This could be explained by the recommendation for patients to keep their eyes closed for at least 20 min (as in Bodin et al. study) or the collection of data during stage 2 NREM sleep (as in Vespa et al. study), when EEG rhythms are dominated by a lower frequency band. For patients after VNS implantation, desynchronization in the higher frequency band is inevitably influenced by stimulation of the VNS device. The only study (Fraschini et al.^17^) to analyze differences in the preoperative interictal awake PLI of 10 patients (32–57 years) between responders and non‐responders found no significant difference, which could be explained by source of undersizing. Because the dynamic change process of EEG activity is adaptive and adjustable to external stimulation, the variability of synchronization biomarkers might reflect the integrity of individual functional brain network connectivity, which responders showed significant desynchronization receiving external stimulation compared to non‐responders. Therefore, our study also proved that pediatric epilepsy patients with higher preoperative synchronicity indexes might be potential VNS responders.

Although clinical information alone is not sufficient for predicting VNS efficacy,^11^, ^12^, ^13^ which was also consistent with our study (the SVM classifier generated from clinical features yielded an accuracy of only 51.4%), we still consider it helpful for improving the accuracy of prediction when integrated with synchronization features. Mithani et al.^22^ also applied multimodal connectomic profiling and SVM classifiers generated from connectomic and clinical features to predict responsiveness to VNS. In our cohort, the SVM classifier generated from integrating clinical and synchronization features revealed an accuracy of 75.7% with an AUC of 0.776, which clearly improved the accuracy of prediction. The principal component coefficient of the selected features in the 10‐fold CV SVM classifier corresponded to the influence of specific features on postoperative VNS efficacy. We could tell from the selected principal component that children with DRE who had fewer current ASMs before implantation, multiple seizure types, and negative MRI findings were more likely to be VNS responders, which also places great emphasis on multidimensional combinations in individualized prediction. Although the accuracy of validation cohort was lower than that of discovery cohort, the deviation was within tolerance, considering the limitation of sample size and heterogeneity of patients in two separate institutions. Furthermore, the application of SVM has been used in outcome prediction for epilepsy surgery,^40^ patient‐specific seizure prediction,^41^ epilepsy diagnosis,^42^ and the identification of the epileptogenic zone.^43^ With further enlargement of sample size and model optimization, the efficacy and generality of SVM prediction model would be ulteriorly improved to meet the need to identify responders to VNS in clinical practice.

Our study had some limitations. First, it was conducted on a relatively small and heterogeneous study population from two separate tertiary epilepsy centers. Although the nature of SVM ensured the accuracy of our results, a more multicenter sample should be included. And we have provided a clinical paradigm and reliable foundation for further research to optimize the prediction algorithm. Second, the age at VNS implantation in our study was heterogeneous (focused mainly between 3 and 9 years old). Limited to sample size in different age groups, age at implantation was not selected as one of the principal components. As further increasing number of different age groups, it might become one of clinical features, which could be adapted in the prediction model. Third, the evaluation of VNS efficacy in our study was bound by 50% reductions in seizure frequency according to the average seizure frequency 6 months before the last follow‐up. Due to the complexity and fluctuation of the epilepsy course in children, it was difficult to completely match the seizure frequency and VNS efficacy. We considered that 75% reduction might be a better critical value for distinguish responders and non‐responders and longer‐term follow‐up would be better for higher sensitivity and specificity. Finally, the biomarker in our study mainly focused on functional connectivity based measures extracted from EEG. Other network or structural connectivity based measures extracted from MRI, DTI and MEG might also be helpful for raising accuracy and precision of prediction model in clinical practice. Hopefully, further study with larger sample size and more network or connectivity based measures could optimize the prediction model to better evaluate VNS efficacy based on our study.

## 5 CONCLUSION

This study established the first prediction model integrating both clinical and synchronization features from baseline scalp EEG for screening VNS responders. With further optimization of the model in the future, we hope to provide an effective, convenient, and individualized method that could be used in clinical practice to identify potential responders before VNS implantation.

## AUTHOR CONTRIBUTIONS

Jiayi Ma, Zhiyan Wang, Luming Li, and Ye Wu involved in conceptualization; Jiayi Ma, Zhiyan Wang, Tungyang Cheng, Yingbing Hu, Xiaoya Qin involved in methodology; Jiayi Ma, Zhiyan Wang, Tungyang Cheng, Yingbing Hu, Xiaoya Qin, Daqi Zha, Wen Wang, Guojing Yu, Qingzhu Liu, Taoyun Ji, Han Xie, Shuang Wang, Zhixian Yang, Xiaoyan Liu, Lixin Cai, Yuwu Jiang, Hongwei Hao and Jing Wang involved in formal analysis and investigation; Jiayi Ma, Zhiyan Wang, Tungyang Cheng and Yingbing Hu wrote the original draft; Luming Li and Ye Wu; Supervision: Luming Li and Ye Wu reviewed and edited the manuscript.

## CONFLICT OF INTEREST

The authors declare that they have no conflict of interest.

## Supporting information


Table S1

Table S2

## Data Availability

Anonymized data and documentation from this study can be made available to qualified investigators upon reasonable request. Such arrangements are subject to standard data sharing agreements.
