# Supplementary material for: A prediction model integrating synchronization biomarkers and clinical features to identify responders to vagus nerve stimulation among pediatric patients with drug‐resistant epilepsy
Source: CNS Neurosci Ther. 2022 Jul 27;28(11):1838–48. doi: 10.1111/cns.13923 (PMC9532924; doi:10.1111/cns.13923)
Supplement: Supplementary file 1 — Table S1 [file CNS-28-1838-s002.docx]

**Supplementary Table 1**. Difference of wPLI and PLI at beta bandwidth (low and high beta bands) between R100, R80 and NR50 groups

|  |  | **NR50s (n=33)** | **R80s (n=28)** | **P value** | **R100s (n=10)** | **P value** |
| --- | --- | --- | --- | --- | --- | --- |
| **PLI** | **low beta** | 0.182±0.007 | 0.184±0.010 | 0.541 | 0.1880±0.016 | 0.273 |
|  | **high beta** | 0.152±0.007 | 0.161±0.020 | 0.006** | 0.1567±0.013 | 0.038* |
| **wPLI** | **low beta** | 0.323±0.020 | 0.334±0.042 | 0.630 | 0.3629±0.058 | 0.063 |
|  | **high beta** | 0.270±0.015 | 0.291±0.036 | 0.054 | 0.3033±0.046 | 0.063 |

Data was showed in mean±standard deviation (SD)

^*^ P<0.05; ^**^ P<0.01; All P value were applied FDR-corrected Mann–Whitney U test.

Abbreviations: PLI = phase lag index; wPLI = weighted phase lag index; R= responders; NR = non-responders.
