# Supplementary material for: A prediction model integrating synchronization biomarkers and clinical features to identify responders to vagus nerve stimulation among pediatric patients with drug‐resistant epilepsy
Source: CNS Neurosci Ther. 2022 Jul 27;28(11):1838–48. doi: 10.1111/cns.13923 (PMC9532924; doi:10.1111/cns.13923)
Supplement: Supplementary file 2 — Table S2 [file CNS-28-1838-s001.docx]

**Supplementary Table 2**. Comparison of PLV, PLI and wPLI at different frequency bands in R50 and NR50 groups among different age ranges

|  |  | **0-3 years** | | **3-9 years** | | **9-16 years** | |
| --- | --- | --- | --- | --- | --- | --- | --- |
|  |  | **R50s (n=5)** | **NR50s (n=3)** | **R50s (n=27)** | **NR50s (n=26)** | **R50s (n=5)** | **NR50s (n=4)** |
| **PLV** | **delta** | 0.489±0.021 | 0.486±0.033 | 0.480±0.035 | 0.481±0.045 | 0.467±0.030 | 0.496±0.092 |
|  | **theta** | 0.434±0.016 | 0.450±0.040 | 0.463±0.054 | 0.444±0.024 | 0.440±0.025 | 0.431±0.027 |
|  | **alpha** | 0.375±0.009 | 0.370±0.035 | 0.387±0.026 | 0.381±0.025 | 0.396±0.031 | 0.369±0.032 |
|  | **low beta** | 0.334±0.032 | 0.322±0.027 | 0.339±0.032 | 0.343±0.031 | 0.354±0.016 | 0.327±0.034 |
|  | **high beta** | 0.329±0.058 | 0.303±0.050 | 0.315±0.049 | 0.327±0.052 | 0.357±0.037 | 0.297±0.047 |
| **PLI** | **delta** | 0.313±0.012 | 0.305±0.019 | 0.307±0.017 | 0.307±0.025 | 0.297±0.012 | 0.344±0.084 |
|  | **theta** | 0.279±0.010 | 0.302±0.031 | 0.298±0.046 | 0.280±0.013 | 0.278±0.006 | 0.274±0.003 |
|  | **alpha** | 0.224±0.005 | 0.220±0.005 | 0.226±0.012 | 0.222±0.010 | 0.229±0.015 | 0.218±0.006 |
|  | **low beta** | 0.179±0.002 | 0.179±0.004 | 0.184±0.010 | 0.182±0.007 | 0.187±0.012 | 0.183±0.005 |
|  | **high beta** | 0.152±0.006 | 0.149±0.000 | 0.162±0.025 | 0.151±0.005 | 0.195±0.036 | 0.158±0.013 |
| **wPLI** | **delta** | 0.049±0.021 | 0.486±0.033 | 0.484±0.025 | 0.483±0.033 | 0.475±0.019 | 0.512±0.081 |
|  | **theta** | 0.499±0.033 | 0.517±0.037 | 0.503±0.042 | 0.487±0.017 | 0.494±0.012 | 0.478±0.004 |
|  | **alpha** | 0.401±0.050 | 0.392±0.013 | 0.389±0.035 | 0.382±0.027 | 0.388±0.023 | 0.369±0.006 |
|  | **low beta** | 0.338±0.056 | 0.327±0.016 | 0.329±0.033 | 0.323±0.022 | 0.336±0.051 | 0.316±0.010 |
|  | **high beta** | 0.293±0.055 | 0.270±0.012 | 0.290±0.035 | 0.269±0.015 | 0.325±0.050 | 0.271±0.019 |

Data was showed in mean±standard deviation (SD)

Abbreviations: PLV = phase locking value; PLI = phase lag index; wPLI = weighted phase lag index; R= responders; NR = non-responders.
